# Adapting temporal preference to scarcity: A role for emotion?

**DOI:** 10.1007/s11166-025-09453-x

**Published:** 2025-06-20

**Authors:** Bastien Blain, Laura K. Globig, Tali Sharot

**Affiliations:** 1 Affective Brain Lab, Department of Experimental Psychology, University College London, London, WC1H 0AP UK; 2 Max Planck UCL Centre for Computational Psychiatry and Ageing Research, University College London, London, UK; 3 Université Panthéon-Sorbonne, CNRS, Centre d’Économie de la Sorbonne, Paris, France; 4 Brain and Cognitive Sciences, Massachusetts Institute of Technology, Cambridge, MA USA; 5 Present Address: Department of Psychology and Neural Science, New York University, New York, NY USA

**Keywords:** Delay Discounting, Temporal Discounting, Emotion, Affect, Income shock

## Abstract

A critical optimization problem is how to distribute resource consumption over time. Humans tend to value immediate rewards over equivalent future rewards—a phenomenon called temporal discounting. Such imbalance can lead to poor health, education, and financial decisions. It is also a hurdle for implementing sustainability policies. A major research goal is to identify factors that influence temporal discounting, so that policymakers could develop interventions to correct for this imbalance. One such factor is available resources; scarcity may increase in temporal discounting. Another potential factor is emotion; negative emotions may lead to high temporal discounting. However, emotion and resources are not independent. For example, losing a large sum of money will lead to negative affect. Here, we take advantage of one of the largest global ‘income shocks’ in history, to tease apart the role of emotion and income on temporal discounting. We tested 1,145 individuals as the market was crashing in late March 2020 and unemployment rising and then retested 200 of those individuals as the market was recovering in June 2020. We found that income shock was strongly related to an increase in delay discounting using cross-sectional and longitudinal data. Importantly, this relationship was independent of the negative impact on affect. These findings suggest that, contrary to wide held assumptions, people directly adapt delay discounting to environmental constraints, without the need for input from the affective system. This independence may be adaptive, as affect is a noisy reflection of environmental constraints, which may lead to suboptimal choice.

**Supplementary Information:**

The online version contains supplementary material available at 10.1007/s11166-025-09453-x.

## Introduction

Achieving desirable long-term outcomes, such as a comfortable retirement, physical health or a sustainable planet, often requires sacrificing consumption now for the benefit of the future. Whether people make such choices depends on how much they value delayed outcomes relative to immediate ones (Samuelson, 1937). While most people discount future rewards relative to immediate rewards (Frederick et al., 2002; Green & Myerson, 2004), the rate at which they do so differs between individuals (Casey et al., 2011; Mischel et al., 1989). This rate, known as the *temporal discount rate*, is higher in individuals who are poor, lack education, and suffer from ill health (de Wit et al., 2007; Sunde et al., 2022; Mischel et al., 2011; Reimers et al., 2009; Schlam et al., 2013). A high discount rate in turn can lead to suboptimal decisions (e.g., lack of saving, unhealthy eating, lack of investment in education) that worsen people’s circumstances further, creating a feedback loop.

Thus, it is crucial to understand how and why difficult life circumstances are associated with temporal discounting, in order to create policies that can break the loop. One suggestion has been that the association between difficult life circumstances and temporal discounting is due to emotion (Haushofer & Fehr, 2014). To put it in Kahneman’s terms, ‘System I’ may be exerting undue influence on financial decisions (Kahneman, 2012; Kahneman & Frederick, 2002). Specifically, poverty, sickness, lack of education all can generate negative mood, which in turn leads to high temporal discounting. This hypothesis is based on studies suggesting that temporal discount rates are sensitive to emotional states. For example, induction of negative affect in laboratory settings led to an increase in temporal discount rate (Lerner et al., 2013; Moore et al., 1976; Schwarz & Pollack, 1977; Seeman & Schwarz, 1974). Perhaps because negative affect is associated with an inability to vividly imagine the future (which is associated with low delay discounting; Lebreton et al., 2013), as observed in depression (Morina et al., 2011).

If negative affect increases delay discount rate, one could potentially decrease individuals’ temporal discount rate using an affective manipulation that impact ‘System I’, even if income remains low. However, difficult life circumstances may lead to high temporal discounting for a number of other reasons, such as low cash flow, regardless of mood. Temporal discounting can be adaptive when resources are scarce, as it leads us to prioritize immediate needs and opportunities to enhance survival rather than focusing on distant, uncertain outcomes. If delay discounting is independent of mood, then decreasing temporal discounting in individuals experiencing difficult life circumstances will likely require a direct change to those circumstances.

**Fig. 1:**
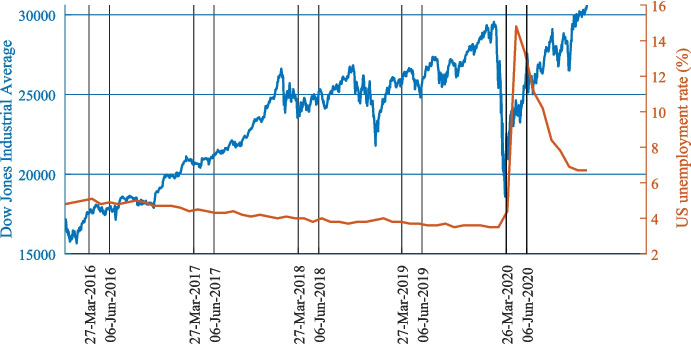
Timing of the Experiment. We tested 1145 individuals on March 26–29, 2020 as the financial markets were plummeting (blue) and the unemployment was sharply increasing (red); we then tested 200 of those individuals again on June 6–7, 2020 as the markets were recovering and the unemployment rate started to decrease (*Bureau of Labor Statistics Data*, n.d.; *INDEXDJX:.DJI—Google Search*, n.d.)

Here, we tease apart the effect of emotion and income on temporal discounting by taking advantage of a naturally occurring, global, income shock due to the market collapse of March 2020. Income shocks (i.e., sudden decrease in income) have been associated with temporal discounting in (Haushofer et al., 2013) and outside the lab (e.g., in farmers in a developing country who experience income shock due to changes in rainfall: Di Falco et al., 2019; Tanaka et al., 2010. In the Western world due to inflation: Dean & Sautmann, 2021; Krupka & Stephens, 2013). The downturn of 2020 began on March 9, with the largest point dive of the US market in history only to be outdone by additional point falls in the weeks that followed. It was also characterized by growing unemployment (Fig. 1). As the situation led to a substantial loss of income for many (not only those invested in the market) it allowed us to investigate the relationship between changes to income, affect and temporal discounting in a large diverse US population (*N* = 1,145). We followed up on a subset of these individuals (*N* = 200) a few months later as the markets were recovering. By recording individuals’ income shock, affective state and delayed discount rates, we were able to characterize the association between income and temporal discounting and reveal whether it is dependent or independent of emotion.

## Methods

We report here how we determined our sample size, all data exclusions, all manipulations, and all measures in the study.

### Participants

We expected an effect size of approximately 0.2, which translates to a sample size of about 200 to achieve 80% power with alpha = 0.05. The expected retention rate of participants on Prolific when tested twice several months apart is about 20% (Kelly & Sharot, 2021). Thus 1,000 participants are required at time 1 to maintain sufficient N at time 2. We added ~ 15% drop out, thus tested 1166 individuals between March 26–29, 2020**,** representative of the US population in terms of age, gender, education, income and ethnicity (see Supplementary Fig. 1A-D). The individuals were residing in 30 US states at the time of testing. They completed an online questionnaire on Prolific Academic. We tested participants’ engagement and attention by asking them to select a particular answer to various “catch trials” throughout the Survey (for example: *Please select ‘strongly disagree’*). Participants who did not select the required response more than once were excluded from analysis (*N* = 21). Thus, data of 1145 participants were analysed (mean age = 44.00, SD = 15.59; females = 52.3%, Democrats = 69%, Republicans = 31%).

We then tested 200 individuals a second time between June 6–7, 2020. Participants from this subsample were representative of the US population in terms of age, gender, education, income, and ethnicity (see Supplementary Fig. 1E-H). Participants who reported positive income shock in June were excluded from that analysis. Thus, data of 184 participants were analysed (age = 44.00, SD = 15.61; females = 52.1%). Participants provided informed consent and received £3.75 for their participation. Ethical approval was provided by the Research Ethics Committee at University College London.

Participants completed an online survey which lasted approximately 30 minutes. In addition to the questions that formed this study, additional information was gathered as part of parallel studies conducted by members of our lab (e.g., Globig et al., 2020). These focused mostly on habits, personality, psychopathology, and other opinions regarding the crisis. We detail the additional information gathered in supplementary material. Below we detail the information gathered via the online survey which is part of the current study.

### Measures

#### Demographics

Participants indicated their age, gender, household income, and education level. Gender was coded as 1 for male and 0 for female.

#### Negative income shock

We measure negative income shock by asking participants to indicate “What is the impact of COVID-19 on your income?” on the following scale: None (1), Extremely low (2), Low (3), Medium (4), High (5), Extremely high (6).

#### Negative affective states

Participants indicated on a continuous visual analogue scale ranging from 0 (not at all) to 100 (very much), the emotion they felt in the last 24 hours (fear, sad, angry). For example, “In the last 24 hours have you felt: fear?”.

#### Delay discounting

We measured delay discounting using the 27-item Kirby questionnaire that has been used with success many times (Kirby et al., 1999). The range of temporal discount rate this questionnaire is sensitive to varies from 0.00001 to 1. For example, a typical question was “Would you prefer: $55 today or $75 in 61 days”.

#### Additional measures at Time II

We added the BIS-BAS questionnaire to test whether income shock affected specifically temporal discount rate or impulsivity more broadly (Beck et al., 1996). Moreover, we wanted to confirm that the results of Time I were specifically due to a negative income shock. Thus, we asked participants not only to answer the question “What is the impact of COVID-19 on your income?” as above, but also to confirm whether this impact on income was positive, negative, or neutral. We included only the participants who indeed experienced a negative income shock which was in fact 92%.

### Data analyses

Delay discounting models were fitted to experimental data by minimizing the negative log likelihood of the predicted choice probability given different model parameters using the fmincon function in MATLAB (Mathworks Inc.). More specifically, choice probability was computed as follows (using the softmax function (Luce, 1958)):

$${P}_{immediate amount}=\frac{1}{1+\text{exp}\left(-\beta \left({U}_{delayed amount}-{U}_{immediate amount}\right)\right)}\;\;\;,$$

where β is a reversed temperature parameter that adjusts the stochasticity of choices.

The utility of each option assumes that reward is discounted by time in a hyperbolic fashion:

$${U}_{delayed amount}=\frac{delayed amount}{1+k\times delay}\;\;\;,$$

where k is a free parameter corresponding to the temporal discount rate. The higher the discount rate, the lower the utility of the discounted reward. Note that for the immediate reward the delay is null and therefore the utility is just equal to the immediate amount. Lower and upper boundaries for temporal discount rate were 0.0001 and 0.25, respectively, corresponding to the range of recoverable values for such a design.

Parameter recovery analyses were performed to ensure that these model parameters were recoverable given the current experimental design.

All the regressions (described in the result section) were estimated using robust regressions (with the MATLAB (Mathworks Inc.) robustfit function), which are designed to be robust to violations heteroscedasticity and outliers. The t-tests performed on the parameters were two tailed. Rank correlations were tested following Spearman approach using the MATLAB ‘corr’ function. Bayes Factor were computed using the bayesFactor toolbox for MATLAB (https://github.com/klabhub/bayesFactor), modified to include robust regressions. This toolbox implements a formula to derived the Bayes factor from the linear model R-squared coefficient of determination of the regression as well as an approximation that we used to avoid infinite values in the integration.

## Results

### Income shock is related to greater delay discounting at Time I & II

One thousand, one hundred forty-five participants (546 males, age = 44 ± 16 (mean ± SD), median yearly income = $52,500; see Supplementary Fig. 1A-D) in the United States completed our online study on March 26–29, 2020. Here, we used a subjective measure of income shock which allows us to account for the fact than an objective change in income can be interpreted differently by different individuals. That is, we wanted to account for the fact that a $1 K income shock can be perceived as large or small by different individuals due, for example, to their net worth, spending habits, income expectations, etc. In other words, people may have unobservable information (Jappelli & Pistaferri, 2010; Kahneman & Tversky, 2013). Thus, participants were asked to indicate the impact of COVID-19 on their income on a 6-point Likert scale from none (1) to extremely high (6). The average reported impact was 3.11(± 0.05 SE) (see Supplementary Fig. 2).

**Fig. 2:**
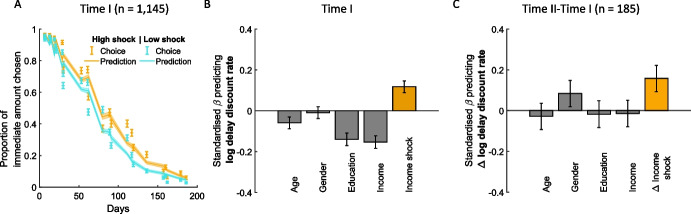
Income shock is related to temporal discounting. **A**: For illustration purposes participants were split into those experiencing high income shock in orange (that is those that reported income shock higher than 3, which is the middle of the scale) and low income shock in cyan ($<$ 3). Displayed are the proportion of trials in which participants selected the immediate reward over the delayed reward (Y axis) as a function of the days associated with the delayed reward. The orange line is above the cyan line indicating that those experiencing higher income shock were more likely to select immediate rewards over delayed rewards for the same temporal delays. The lines represent the prediction of the hyperbolic model and dots represent observed data. Error bars and shaded areas represents the standard error of the mean. *N* = 1,145. **B**: There was a strong relationship between income shock and the log temporal discount rate in a model controlling for all demographic variables. In addition, greater temporal discount rate was also related to less education, low income and a trend relationship with age. Figures show standardized coefficients from a linear model predicting the logged temporal discount rates. **C**: Temporal changes in discount rate are related to temporal changes in income shock, here illustrated when controlling for demographics variables. *N* = 184. Gender: 1 corresponds to male and 0 to female. Error bars correspond to the standard error of the mean. * *p* < 0.05, ***P < 0.001

To test the relationship between income shock and delay discounting, participants completed 27 choice trials (Kirby et al., 1999), each requiring them to choose between a smaller, immediate amount of money (e.g., $25 today) versus a larger delayed amount of money (e.g., $35 in 25 days; see methods for details). We then estimated the discount rate for each participant by fitting a simple hyperbolic model to the intertemporal choice data. More specifically, immediate amount choices were fitted using a standard softmax function of the utility (U_i_) difference between the two options, which provides the probability of choosing the immediate reward (Luce, 1958). discount rate. The model provided a good fit to participants’ choices (pseudo-R^2^ = 0.69 ± 0.0059, mean ± SE; balanced accuracy = 0.87 ± 0.004, mean ± SE; see Fig. 2A and methods). Temporal discount rates were log transformed to ensure that they follow a normal distribution.

For the cross-section analysis, we then estimated a linear model with log discount rate as the dependent variable and income shock as the independent variable controlling for all demographics (age, gender, education, and income). More specifically, we estimated the following equation:

$$\text{log}\ {\left(k\right)}_{i1}= {\beta }_{0}+ {\beta }_{1}Ag{e}_{i1}+ {\beta }_{2}Gende{r}_{i1}+ {\beta }_{3}Educatio{n}_{i1}+ {\beta }_{4}Incom{e}_{i1}+{\beta }_{5} IncomeShoc{k}_{i1}.$$

The subscript i refers to each individual, while 1 refers to Time I. In compliance with the policy of the *Journal of Risk and Uncertainty *ordinal scores for the Income Shock likert scale were converted as follows: None (1), Extremely low (2), Low (3) were converted to 0; Medium (4), High (5), Extremely high (6) were converted to 1. Estimating this model revealed a significant relationship between income shock and log discount rate (median k = 0.0137 ± 0.0016 (M ± SE); β = 0.12 ± 0.03, t_1139_ = 4.1, *p* < 10^–4^; see Fig. 2B), with greater shock leading to greater discount rates. The same effect is observed when not controlling for demographics (β = 0.15 ± 0.029, t_1143_ = 5.0, *p* < 10^–6^). Income shock effects (standardized β coefficients ~ 0.10–0.20) fall in the small-to-moderate range as per Cohen’s benchmark (Nieminen, 2022).

We also replicate previous findings (de Wit et al., 2007; Sunde et al., 2022; Mischel et al., 2011; Reimers et al., 2009; Schlam et al., 2013), showing that greater delay discount rate is related to less education (β = −0.14 ± 0.03, t_1139_ = −4.6, *p* < 10^–5^) and lower income (β = −0.15 ± 0.03, t_1139_ = −5.1, *p* < 10^–6^), and in younger age (β = −0.06 ± 0.03, t_1139_ = −2.0, p = 0.04). There was no relationship with gender (β = −0.009 ± 0.03, t_1139_ = −0.33, *p* = 0.74).

Two hundred of the above participants (103 males, age = 47 ± 15 (mean ± SD), median yearly income = $52,500) completed the study again on June 6–7, 2020 as markets were recovering (see Fig. 1). Collecting data across two timepoints allowed us to (1) test for the replicability of the results observed at Time I in an environment which was slightly different, and (2) to test for the effect of income shock longitudinally.

As expected, income shock was significant lower in June (2.9 ± 0.11, mean ± SE) relative to March (3.2 ± 0.11, mean ± SE; difference = 0.27 ± 0.1, t_183_ = 2.6, *p* < 0.01; Supplementary Fig. 2D). There was still a wide range of responses in June, with participants reporting income shock from none (0) to extremely high (6) (see Supplementary Fig. 2A and B). This variance of income shock across individuals and timepoints allowed us to study the effect of income shock on delay discounting cross-sectionally and longitudinally.

At the second timepoint we asked participants whether the income shock was positive or negative. The vast majority of the participants (92%, *N* = 184) reported a negative shock or no shock with only 8% (*n* = 16) reporting a positive shock. We excluded data from the participants who reported the positive shock as our research question was focused on negative income shock. All results detailed below remain the same regardless of whether these participants were included or not, unless otherwise specified.

Within this smaller subset the delay discounting model still provided a good fit to participants’ choices (Time I: pseudo-R^2^ = 0.692 ± 0.016 (mean ± SE); balanced accuracy = 0.883 ± 0.008 (mean ± SE); Time II: pseudo-R^2^ = 0.875 ± 0.017; balanced accuracy = 0.941 ± 0.006; see Supplementary Fig. 3A & B and methods). As above, temporal discount rates were log transformed to ensure that they follow a normal distribution.

In the frame of the cross-section analysis, we then estimated a linear model with log discount rate as the dependent variable and income shock as the independent variable controlling for all demographics (age, gender, education, and income) in this subset of participants (*N* = 184) in each timepoint as done previously. Note that in compliance with the policy of the* Journal of Risk and Uncertainty* ordinal scores for the Income Shock likert scale were converted as follows: None (1), Extremely low (2), Low (3) were converted to 0; Medium (4), High (5), Extremely high (6) were converted to 1. This revealed a significant relationship between income shock and log discount rate at both timepoints (Time I: median k = 0.0125 ± 0.0033 (M ± SE); β = 0.20 ± 0.068, t_178_ = 2.9, p = 0.004; Time II: median k = 0.0137 ± 0.0041(M ± SE); β = 0.20 ± 0.076, t_178_ = 2.7, *p* < 0.008; see Supplementary Fig. 3A, B for illustration), with income shock leading to greater discount rates. The same effect is observed when not controlling for demographics (Time I: β = 0.17 ± 0.070, t_182_ = 2.4, *p* = 0.017; Time II: β = 0.23 ± 0.077, t_182_ = 3.0, *p* < 0.003). Thus, the results were replicated at time 2. Income shock effects (standardized β coefficients ~ 0.10–0.20) fall in the small-to-moderate range as per Cohen’s benchmark (Nieminen, 2022).

Next, in a panel analysis, we examined whether changes in income shock across time were related to temporal changes in delay discounting. More specifically, we estimated the following equation:

$$\text{log}\ {\left(k\right)}_{\Delta t}= {\beta }_{0}+ {\beta }_{1}Ag{e}_{j1}+ {\beta }_{2}Gende{r}_{j1}+ {\beta }_{3}Educatio{n}_{j1}+ {\beta }_{4}Incom{e}_{j1}+ {\beta }_{5} IncomeShoc{k}_{j\Delta t}.$$

The subscript *j* refers to each individual from the subset, while ∆t corresponds to the difference of the variable between Time II and Time I. Indeed, we found that the greater the increase in income shock an individual experienced, the greater the increase in temporal discount rates the individual displayed correcting for demographics (β = 0.16 ± 0.064, t_178_ = 2.4, *p* = 0.015) or not (β = 0.14 ± 0.059, t_182_ = 2.3, *p* = 0.022; Fig. 2C). Including participants with positive income shock leads to the same results when correcting for demographics (β = 0.14 ± 0.06, t_194_ = 2.2, *p* = 0.027), as well as when not correcting for demographics (β = 0.12 ± 0.06, t_198_ = 2.1, *p* = 0.039). Income shock effects (standardized β coefficients ~ 0.10–0.20) fall in the small-to-moderate range as per Cohen’s benchmark (Nieminen, 2022).

Thus far, we have shown that income shock led to a highly significant increase in temporal discount rates across participants (cross-section analysis), and that temporal changes in income shock were linked to temporal changes in delay discounting (panel analysis), in support of our prediction. Given that income shock was due to an external event (a pandemic) it is more likely that income shock led to greater delay discounting than the other way around.

### Negative affect is related to greater temporal discounting within the full sample (Time I)

Participants reported their emotions (fear, sadness, anger) in the last 24 hours on a continuous scale ranging from 0 (none) to 100 (extremely high). We found that negative emotions were strongly correlated with one another (fear, sadness, anger; all Spearman ρ > 0.5, all *p* < 10^–20^; see Supplementary Fig. 4A). We therefore averaged the ratings for negative emotions as a measure of ‘negative affective state’. The mean response in negative affect was 43 ± 25 (Mean ± SD), suggesting that participants were experiencing negative emotions (see Supplementary Fig. 4B). There was large individual variability in participants’ negative affect with individual averaged ratings varying from 0 to 95 (100 being the maximum). In compliance with the policy of the *Journal of Risk and Uncertainty*, rating for each emotion were converted as follows: values strictly lower than 33.33 were converted to 0 (low), values strictly higher than 33.33 and lower than 66.66 were converted to 0.5, whereas values strictly higher than 66.66 were converted to 1.

To test whether negative affect was related to temporal discount rates in a cross-sectional analysis, we conducted a linear regression with log delay discounting as the dependent measure and negative affect as the independent measure, controlling for demographics as above. More specifically, we estimated the equation below:

$$\text{log}\ {\left(k\right)}_{i1}= {\beta }_{0}+ {\beta }_{1}Ag{e}_{i1}+ {\beta }_{2}Gende{r}_{i1}+ {\beta }_{3}Educatio{n}_{i1}+ {\beta }_{4}Incom{e}_{i1}+{\beta }_{5}Incom{eSchock}_{i1}+ {\beta }_{6} NegativeAffec{t}_{i1}.$$

The subscript i refers to each individual, and 1 to Time I.

**Fig. 3:**
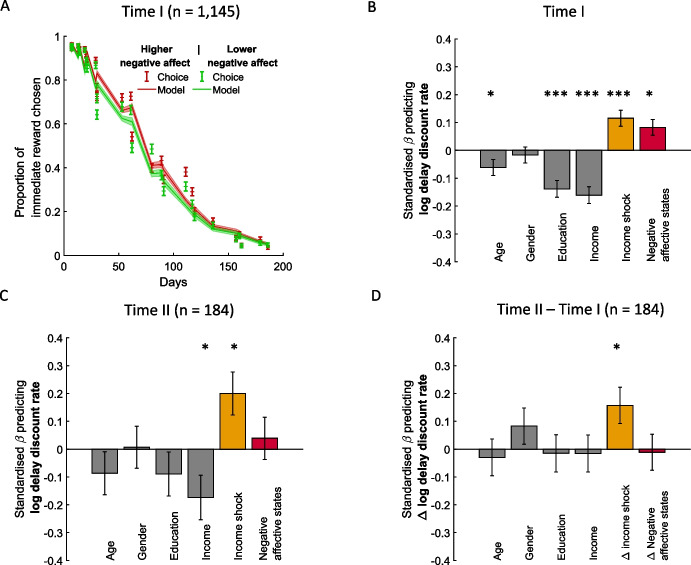
Temporal discounting is related to negative affect. **A**: For illustration purposes participants were split to those experiencing high negative affective state in red ($\ge$ 50) and low negative affective state in green ($<$ 50). Displayed are the proportion of trials in which participants selected the immediate reward over the delayed reward (Y axis) as a function of the days associated with the delayed reward. The lines represent the prediction of the hyperbolic model and dots represent observed data. Error bars and shaded areas represents the standard error of the mean. **B**: Figure shows standardized coefficients from a linear model predicting the logged temporal discount rates when both affect and income shock compete for variance, at Time I. Logged temporal discount rate was related to greater negative affect and greater income shock, suggesting both variables independently influence temporal discounting. Gender: 1 corresponds to male and 0 to female. *N* = 1,145. **C**: Figure is similar to B except that it corresponds to the subsample at Time II (*N* = 184.). Logged temporal discount rate was related to income shock, replicating results at Time I; yet, it was no longer related to negative affective states. **D**: Temporal changes in discount rate are not related to temporal changes in negative affective states, but are related to income shock. Here illustrated when controlling for demographics variables. *N* = 184. Error bars correspond to the standard error of the mean. *P < 0.05, ***P < 0.0001

Replicating previous reports (e.g., Lerner et al., 2013) we found that greater negative emotion was related to greater delay discount rate (β = 0.082 ± 0.028, t_1138_ = 2.9, *p* = 0.0037; see Fig. 3A for illustration), not controlling for demographics led to similar results: β = 0.062 ± 0.029, t_1142_ = 2.11, *p* = 0.035). Emotion effects (standardized β coefficients < 0.10) fall below the small range as per Cohen’s benchmark (Nieminen, 2022).

Moreover, the effect of income shock on log delayed discounting remained positive when controlling for negative affective states and for demographics (β = 0.12 ± 0.03, t_1138_ = 4.0, *p* < 10^–4^) or not (β = 0.15 ± 0.03, t_1142_ = 5.0, *p* < 10^–6^) and income shock and emotions were not significantly correlated with one another (Pearson r = 0.025, *p* = 0.40).

While income shock and emotions were measured on different scales, they are both standardised. Thus, the magnitude of the Beta represents how many standard deviations the log discount rate changes for one standard deviation change in each predictor. In this relative terms, income shock has a larger effect ($\Delta\upbeta =0.08,$ t-test comparing the standardized betas: ${t}_{1143}=2.0, p=0.04$). This provides the first clue that the effects of income shock on temporal discounting cannot be explained solely by refereeing to changes in emotion. We formally test this notion later using mediation models.

Within the subset of participants (*N* = 184) whose data was collected at both Time I and Time II, delayed discount rate was still associated with income shock (Time I, controlling for demographics: β = 0.19 ± 0.07, t_177_ = 2.6, *p* = 0.011; not controlling for demographics:; Time II, controlling for demographics: β = 0.20 ± 0.07, t_177_ = 2.6, *p* = 0.010; not controlling for demographics: β = 0.23 ± 0.08, t_181_ = 2.9, *p* = 0.004; Fig. 3C), but not with affective state (Time I; β = 0.07 ± 0.07, t_177_ = 1.0, *p* = 0.33, Bayes Factor (BF) = 0.1; not controlling for demographics: β = 0.05 ± 0.07, t_182_ = 0.7, *p* = 0.48, BF = 0.06; Time II: β = 0.04 ± 0.08, t_177_ = 0.51, *p* = 0.61, BF = 0.07; not controlling for demographics β = 0.05 ± 0.08, t_181_ = 0.6, *p* = 0.56, BF = 0.06; Supplementary Fig. 5A and B for illustration). Neither did the temporal change in delay discounting relate to the temporal change in negative affect in this subsample (controlling for demographics: β = −0.048 ± 0.06, t_178_ = −0.7, *p* = 0.45; see Fig. 3D; or not β = −0.10 ± 0.06, t_182_ = −1.7, *p* = 0.095 –a trend in the opposite direction). Bayes factor analysis supported the conclusion that affective state was not associated with temporal discounting in the subsample. Specifically, we compute the Bayes factor between the full model and the nested model without affective states. Bayes factors smaller than 1/3 suggests evidence for the null hypothesis (Andraszewicz et al., 2015). The Bayes factor was indeed smaller than 1/3, suggesting evidence for the null hypothesis that negative affect was not relate to temporal discounting (controlling for demographics Time I: Bayes Factor (BF) = 0.1; not controlling for demographics: BF = 0.17; Time II: controlling for demographics: BF = 0.07; not controlling for demographics: BF = 0.17) and that the temporal change in negative affect was not associated with the temporal changes in delay discounting (controlling for demographics: BF = 0.06; not controlling for demographics: BF = 0.12). The reason for the difference in results regarding the association between affect and temporal discounting between the small subsample and the full sample are unclear, but seem to indicate that the relationship is not as strong as the one between income change and temporal discounting, which is observable even when looking at smaller samples.

Thus far, our results suggest that income shock was linked to delay discount rate across participants in both the full sample and the subset, as well as longitudinally, whereas the link between negative affect and delay discounting was only observe across participants in the full sample, possibly because the effect size for the latter was smaller and required a larger N. Indeed, the impact of income shock on delay discount rate was larger than that of negative affect.

### The relationship between income shock and delay discounting is independent of affect

We next ask whether the relationship between income shock and temporal discounting was (in)dependent from affect. To answer this question, we conducted a formal mediation to test whether negative affective state was mediating the effect of income shock on temporal discount rate. Using a Sobel test on the product of the weight between income shock and affective state and the weight from affective state to temporal discount rate (denoted alpha and beta respectively), we found that negative affective state did not mediate the effect of income shock on temporal delay discounting regardless of whether we controlled for demographics (Sobel test on alpha*beta = 0.003 ± 0.003, t_1138_ = 0.99, *p* = 0.32) or not (Sobel test on alpha*beta = 0.002 ± 0.002, t_1142_ = 0.94, *p* = 0.35).

Taken together, these results suggest that the relationship between income shock and delay discounting is not mediated by affect.

## Discussion

In this study, we examined how a naturally occurring income shock, which impacted a large diverse population, influenced temporal discount rate, affect, and their interactions. In particular, we looked at the effect of a real life-threatening situation—the 2020 pandemic—and its actual economic impact at two timepoints. We tested a large representative population in the US in late March 2020 when markets were crushing, and in early June 2020 when markets were recovering.

We found that this naturally occurring income shock was related to larger temporal discount rates in a diverse population. In particular, individuals reporting greater income shock exhibited higher temporal discount rates. Moreover, greater negative change in income shock within an individual over time was related to greater increase in discount rate over time. These results generalize previous work, in which decrease in income was measured in Ethiopian farmers or manipulated in the lab (Di Falco et al., 2019; Haushofer et al., 2013; Tanaka et al., 2010), to a real-life event impacting a diverse population.

We were specifically interested in examining whether both income shock and negative affect influenced temporal discounting, and if they do so independently. To study this, we first examined for a relationship between negative affect and delay discounting. Using a real-life threatening situation that impacted a large diverse population, we observe a link between negative affect and greater temporal discounting, in line with studies in which affect was manipulated in the lab (e.g., Lerner et al., 2013). Importantly, however, a mediation analysis revealed that negative affect was not mediating the relationship between income shock and temporal discount rate. Moreover, when both negative affect and income shock were entered into one model to compete for variance, they both predicted temporal discount rate. This suggest that these two factors independently impact financial temporal discount rates. That is, changes in affect that are not directly caused by income changes likely alter temporal discount rates and equivalently changes in income that are not accompanied by an emotional response can alter discount rates. Income shock effects (standardized β coefficients ~ 0.12–0.20) fall in the small-to-moderate range as per Cohen’s benchmark (Nieminen, 2022). Negative affect has a smaller effect (~ 0.08) and loses significance in the longitudinal analysis, suggesting it may be less robust. Income shock's effect (~ 0.12–0.20) is comparable to the effects of education (−0.14) and income (−0.15), suggesting it is in the same order of magnitude as well-established predictors of discounting. Note, that we tested temporal discounting in the domain of finance. Future studies are needed to examine whether similar results are observed for temporal choices in other domains such as food or other consumables (Mellis et al., 2018).

Many scholars have suggested that temporal discounting arises partly because the future is inherently more uncertain than the present (e.g., Sozou, 1998; Dasgupta & Maskin, 2005; Halevy, 2008; Epper et al., 2009; Saito, 2011; Andreoni & Sprenger, 2012; Sharot et al., 2011). This allows for flexible beliefs regarding future outcomes, which lead to decisions consistent with temporal discounting. For example, when faced with a decision between consuming $10 K now or in one year, a person may decide to consume now because they assume they will be able to gain additional $10 K by next year. Alternatively, they may decide to consume now as they are concerned the money will be taken away from them in the coming months. In either case, uncertainty regarding the future leads to choices to consume now.

Together, our results suggest that income shock leads to an increase in temporal discount rate independently of negative affect. Moreover, the impact of income shock on temporal discounting was greater than of negative affect. It seems that people directly adapt delay discounting to environmental constraints, without the need of input from the affective system. This may be adaptive as affect is a noisy reflection of environmental constraints, and as such could introduce noise to the decision problem leading to suboptimal behaviour. Instead of being influenced by negative affective state, delay discounting could be primarily determined by liquidity constraints.

The increase in temporal discounting following an income shock has potential implications in terms of policy. If individuals experiencing financial strain exhibit higher temporal discounting, they may be less inclined to support sustainability initiatives that require upfront costs but yield benefits in the distant future. This has direct consequences for public acceptance of policies that require short-term sacrifices for long-term gains, such as climate change mitigation policies or social investment programs. This aligns with prior work showing that economic downturns can reduce public prioritization of environmental issues (Motel, 2014). Therefore, policies aimed at enhancing sustainability may be more widely accepted after income shock is resolved.

## Supplementary Information

Below is the link to the electronic supplementary material.

Supplementary file1 (DOCX 1675 KB)

## Data Availability

Data and analysis code are available at: https://github.com/affective-brain-lab/Adapting-temporal-preference-to-scarcity
