# Supplementary material for: Adapting temporal preference to scarcity: A role for emotion?
Source: J Risk Uncertain. 2025 Jun 20;71(1):93–109. doi: 10.1007/s11166-025-09453-x (PMC12373532; doi:10.1007/s11166-025-09453-x)
Supplement: Supplementary file 1 — Supplementary file1 (DOCX 1675 KB) [file 11166_2025_9453_MOESM1_ESM.docx]

**Supplementary information**

**Adapting temporal preference to scarcity: a role for emotion?**

Bastien Blain^1,2,3,^*, Laura Globig^1,2^ & Tali Sharot^1,2,4^*

1 Affective Brain Lab, Department of Experimental Psychology, University College London, London, WC1H 0AP, UK.

2 Max Planck UCL Centre for Computational Psychiatry and Ageing Research, University College London, London, United Kingdom

3 Sorbonne Economics Centre, Pantheon-Sorbonne University, Paris, 75005, France

4 Brain and Cognitive Sciences, Massachusetts Institute of Technology, Cambridge, MA, USA.

*Correspondence to: [b.blain@ucl.ac.uk](mailto:b.blain@ucl.ac.uk), [t.sharot@ucl.ac.uk](mailto:t.sharot@ucl.ac.uk)

**Content**

**Supplementary figure 1. Overview of demographics.**

**Supplementary figure 2. A. Income shock, whole dataset at time 1 (N = 1,145).**

**Supplementary figure 3. Income shock is related to temporal discount rates in the subset of participants at time I and time II.**

**Supplementary figure 4. Affective states.**

**Supplementary figure 5. Temporal discount rate is related to negative affect in the**

**subset of participants in Time 1 but not Time 2.**

**Additional information obtained in questionnaire at time 1**


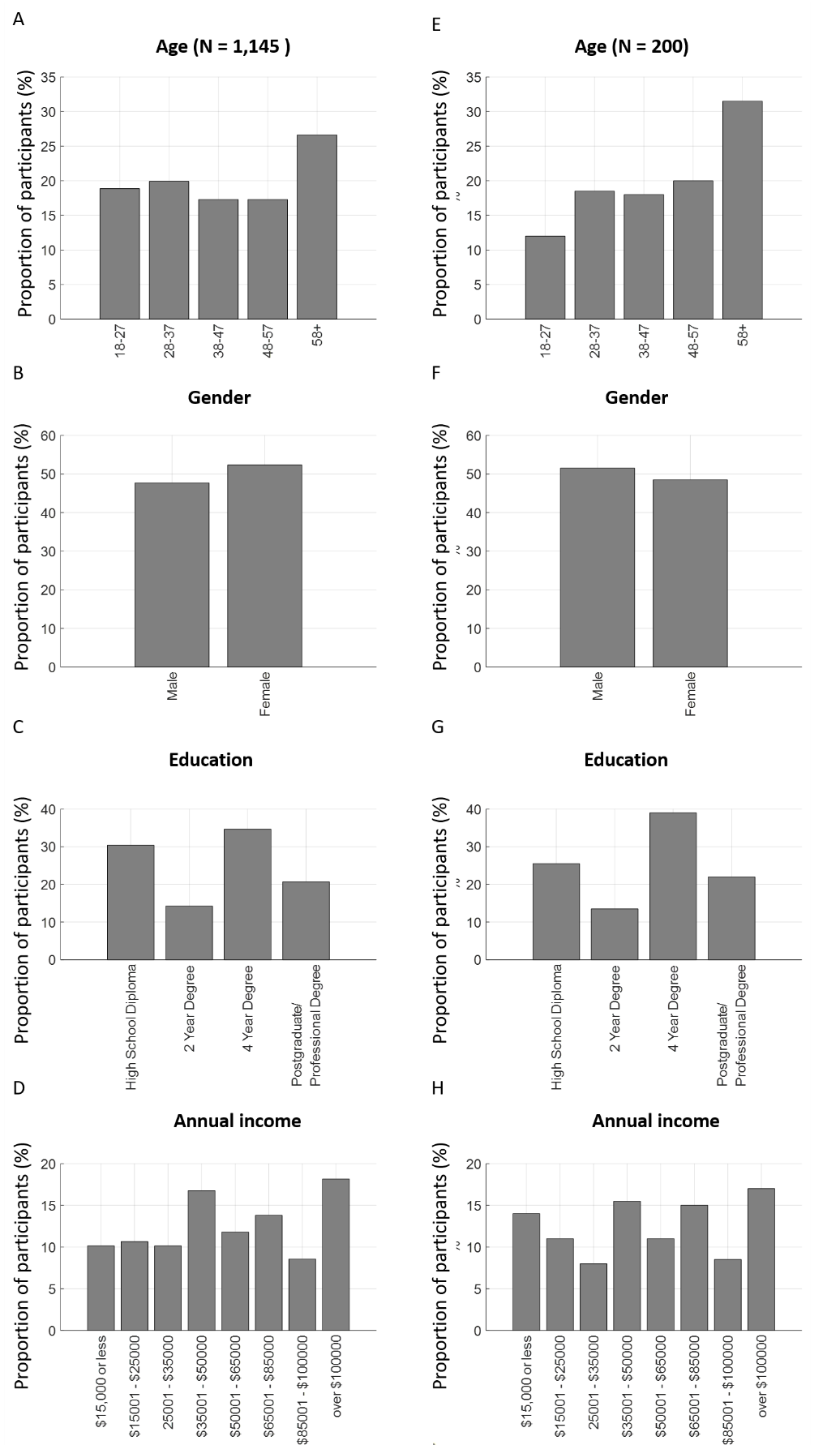


**Supplementary figure 1. Overview of demographics.** Sample (N = 1145) is representative of the US population in terms of **(a)** Age, **(b)** Gender, and **(c)** Education, and **(d)** Income, as well as the subsample (N = 200, **e-h**). Participants resided in 30 US States at time of testing.


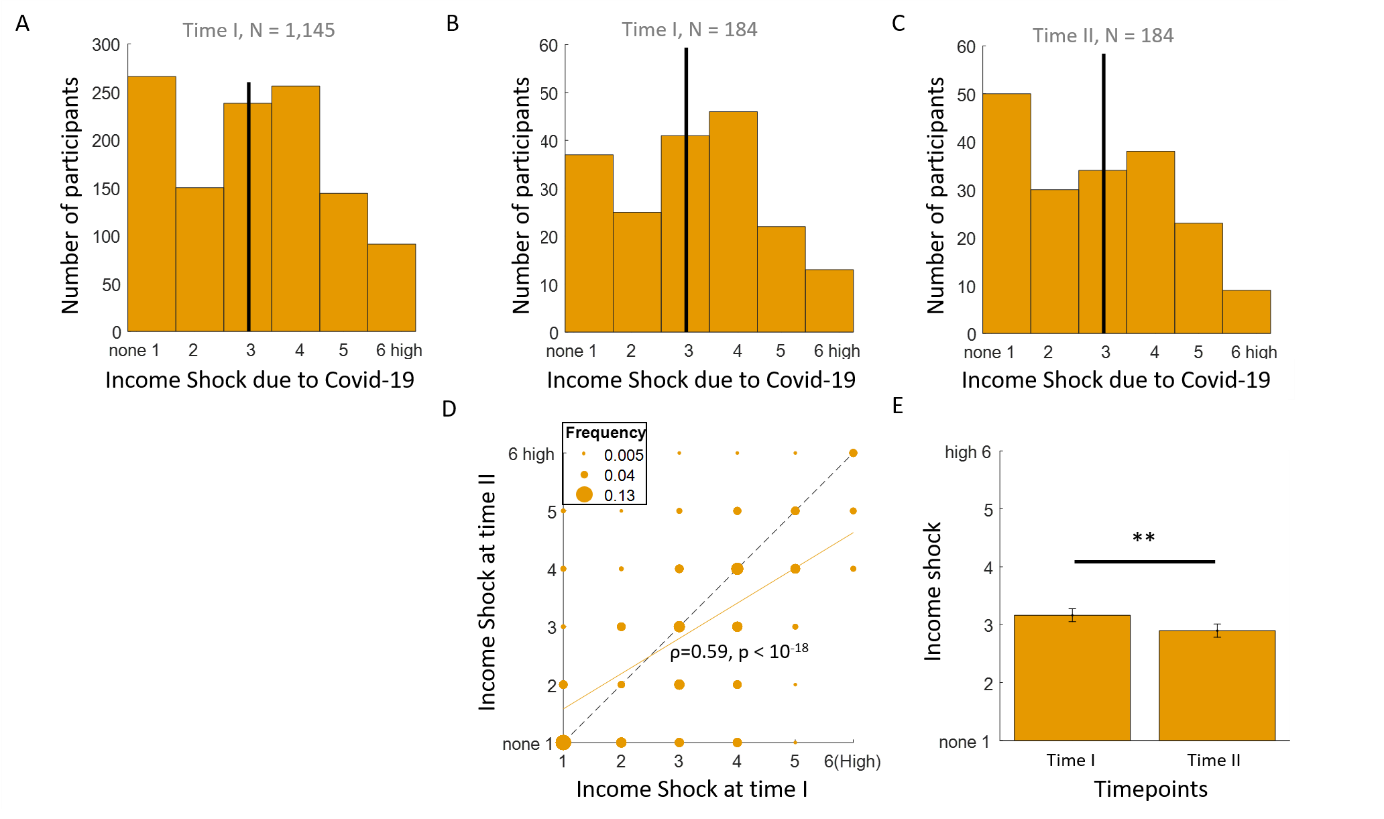


**Supplementary figure 2. A. Income shock, whole dataset at time 1 (N = 1,145).** Distribution of the reported income shock on a scale from 1 (no income shock) via 3 (moderate income shock) to 6 (high income shock). The black vertical lines correspond to the median (M = 3).

**B-E. Income shock in the data subset (N = 182)**. **B & C.** Distribution of the reported income shock on a scale from 1 (no income shock) via 3 (moderate income shock) to 6 (high income shock). The black vertical lines correspond to the median (M = 3). **D**. Temporal correlation of income shock across participants. Income shock at time I is represented on the x axis, while income shock at time II is represented on the y axis. Each dot represents the proportion of participants at the coordinates. The legend represents the sizes corresponding to the minimal, the median and the maximal frequency, respectively. Dashed line the equality line. **E**. average income shock decreased between each timepoint (March 2020 and June 2020 respectively). Error bars correspond to the standard error of the mean. **P<0.01


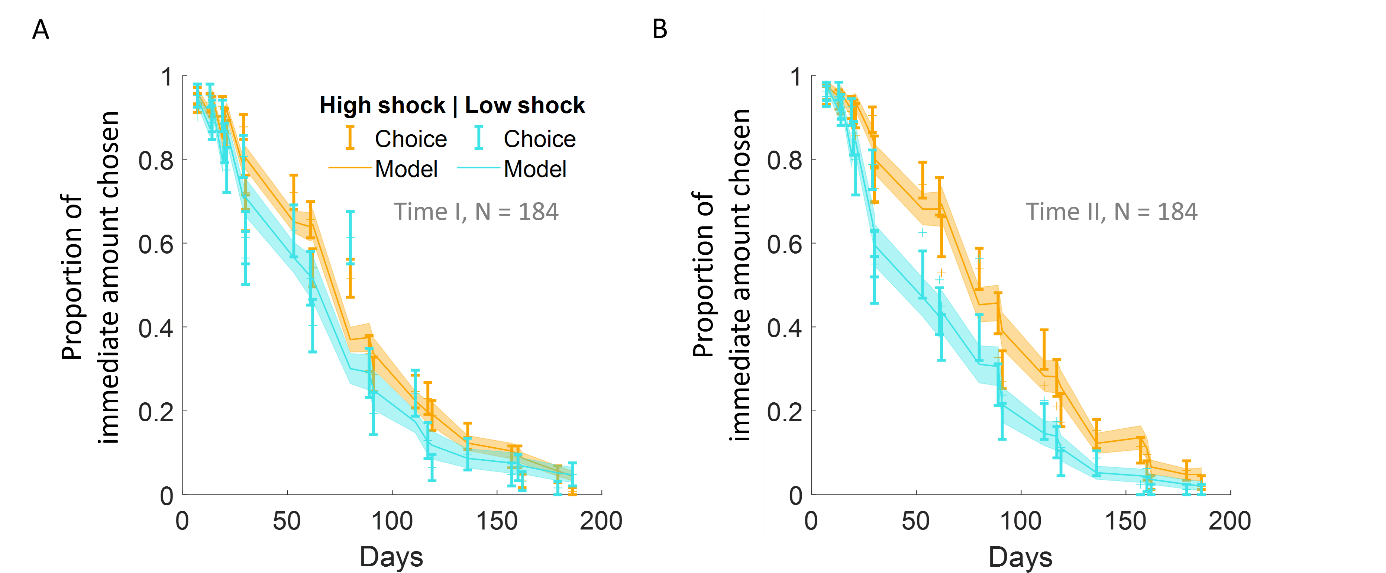


**Supplementary figure 3. Income shock is related to temporal discount rates in the subset of participants at time I and time II.** The left panel corresponds to data collected at time I, and the right panel to data collected at time II. **A&B**: For illustration purposes participants were split to those experiencing high income shock in orange (that is those that reported income shock higher than 3, which is the middle of the scale) and low income shock in cyan ($<$3). Displayed are the proportion of trials in which participants selected the immediate reward over the delayed reward (Y axis) as a function of the days associated with the delayed reward. The orange line is above the cyan line indicating that those experiencing higher income shock were more likely to select immediate rewards over delayed rewards for the same delays. The lines represent the prediction of the hyperbolic model and dots represent observed data. Error bars and shaded areas represents the standard error of the mean**.**


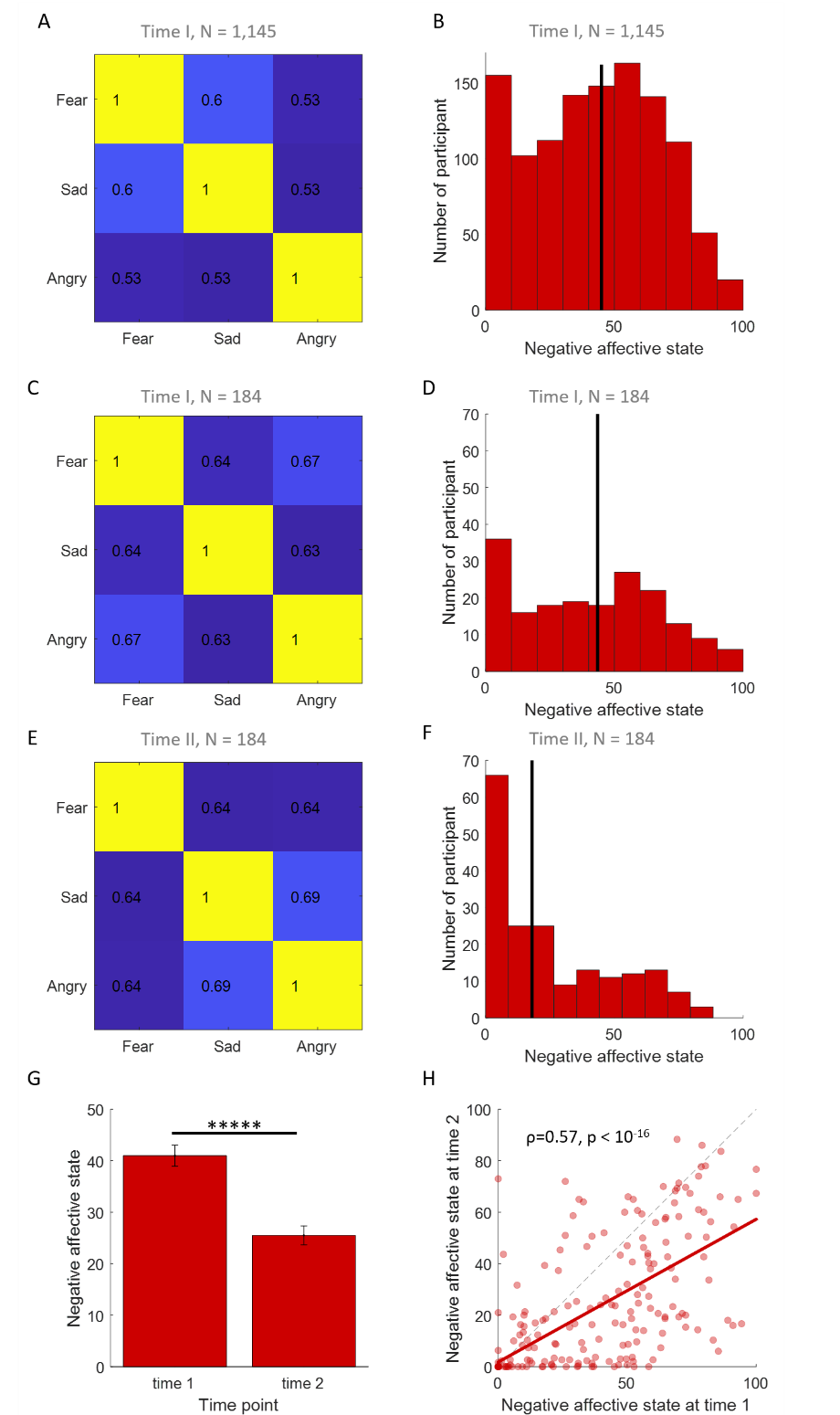


**Supplementary figure 4. Affective states. A, C, E:** Self-reported negative emotions for the whole dataset at time I (**A**), for the subsample at time I (**C**), and for the subsample at time II (**E**) are correlated with each other (all Spearman ρ are displayed, all p < 10^-20^). These were averaged, creating a single construct which we refer to as negative affective state. **B, D, F:** Distribution of negative affective state in the population for the whole dataset at time I (**B**), for the subsample at time I (**D**), and for the subsample at time II (**F**). The vertical black line corresponds to the median. **G**: Average negative affective state was reduced over time. Error bars correspond to the standard error of the mean. **H**: Most participants experienced more negative affective states at time 1 than at time 2 although both were correlated, with approximately 70 participants who experienced no or almost no negative affective state at Time 2. *****p < 10^-14^


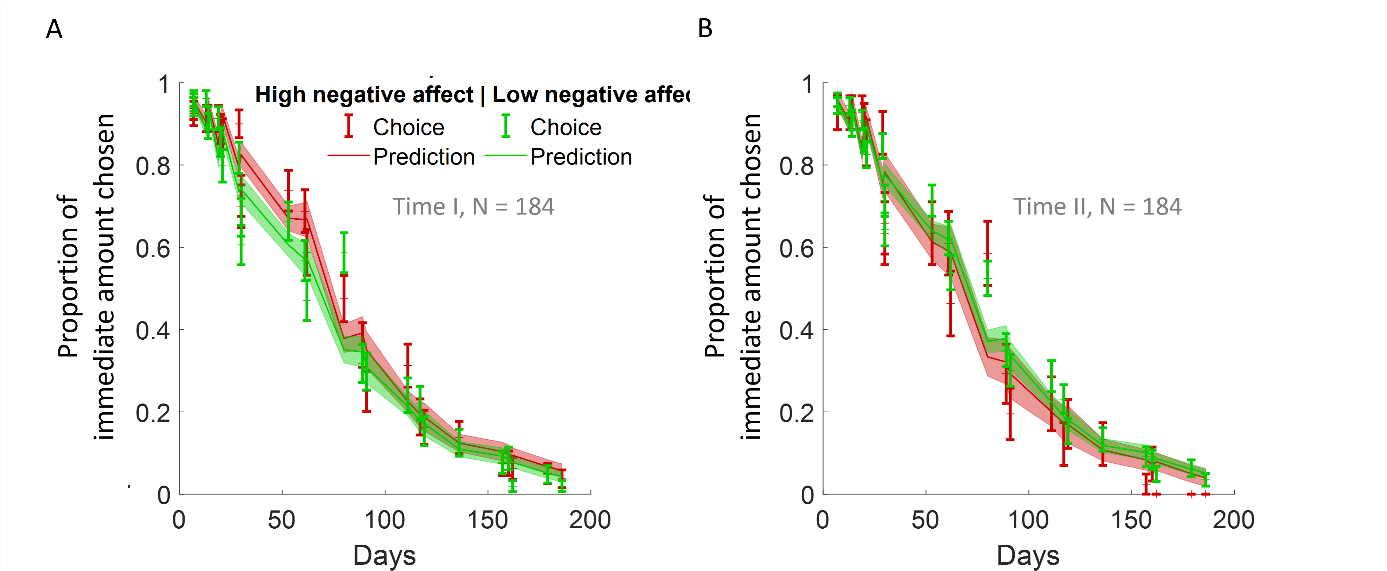


**Supplementary figure 5. Temporal discount rate is related to negative affect in the subset of participants in Time 1 but not Time 2. A:** data collected at time 1 of the subset. **B:** data collected at time 2. For illustration purposes participants were median split into those experiencing high negative affective state in red ($\geq$50) and low negative affective state in green ($<$ 50). Displayed are the proportion of trials in which participants selected the immediate reward over the delayed reward (Y axis) as a function of the days associated with the delayed reward (X axis). The lines represent the prediction of the hyperbolic model and dots represent observed data. Error bars and shaded areas represents the standard error of the mean.

**Additional information obtained in questionnaire at time 1** (which are part of parallel studies conducted in our lab):

**Behavioural Changes.** To assess behavioural changes participants were asked to indicate the frequency of face-to-face interaction, online/telephone interaction, physical activity, outdoor activity, visiting places of religious worship before and after the restrictions.

**Addictive behaviours.** Participants reported frequency of habitudinal and addictive behaviours including smoking, alcohol consumption, gambling, eating before and after the restrictions.

**Psychopathology.** Participants completed the Obsessive-Compulsive Inventory – Revised (OCI-R, (Foa et al., 1998), Patient Health Questionnaire (PHQ-9, Kroenke et al., 2001) Apathy Evaluation Scale (AES, (Mann, 1990).

**Psychosocial Questionnaires:** Participants completed a series of psychosocial questionnaires assessing empathic concern (Davis, 1983), resilience (Smith et al., 2008), narcissism (Leckelt et al., 2018), risk-taking propensity (GRiPS, (Zhang et al., 2019).

**Stress Coping.** Participants responded on a 5-point Likert Scale from 1 (strongly disagree) to 5 (strongly agree) to the following items: 1) The effects of stress are negative and should be avoided. 2) The effects of stress are positive and should be utilized.

**Health Anxiety.** Participants were asked to indicate health anxiety on a 5-point Likert Scale from 1 (very inaccurate) to 5 (very accurate): Often I am concerned about diseases I might have.

**Non-Conformity.** Participants were asked to indicate conformity on a 5-point Likert Scale from 1 (very strong disagreement) to 5 (very strong agreement): I prefer to make my own way in life rather than find and follow.

**Social Support and Connectedness.** Participants were asked to indicate on a 7-point Likert Scale from 1 (strongly disagree) to 7 (strongly agree): My friends/family give me the support I need. We presented participants with a modified version of the “inclusion of others in the self” scale (Aron et al., 1991).

**Anxiety about possible implications of COVID-19.** Participants indicated how anxious they were on a scale of 0 (not at all) to 100 (very much): a) “Are you anxious about your own health in light of COVID-19?”; b) “Are you anxious about the health of your loved ones in light of COVID-19?”; c) “Are you anxious about dealing with lockdown in your area?”; d) “Are you anxious about the consequence to your income/savings in light of COVID-19?”; e) “Are you anxious about home-schooling in light of COVID-19?”; f) “Are you anxious about not being able to exercise?”; g) “Are you anxious about not having access to food/medicine/other supplies?”; h) “Are you anxious about not being able to socialize?”

**Anxiety.** We assessed general anxiety using the short version of the State-Trait Anxiety Inventory (STAI(Marteau & Bekker, 1992)

**Happiness.** Participants were asked two question to assess their happiness: (i) “Taken all together, how happy are you with your life these days? Mark your rating relative to the least and most happy time of your life.” Participants were asked to respond on a continuous visual analogue scale ranging from 0 (least happy time of your life) to 100 (most happy time of your life). (ii) “Think about right now. How happy are you at this moment?” to respond on a continuous visual analogue scale ranging from 0 (very unhappy) to 100 (very happy).

**Relative Private Optimism.** Participants were asked to indicate: “Relative to others of your age and gender do you think you are less/more likely to get COVID-19?” on a scale from 1 (much less likely) to 5 (much more likely). Assessing one’s risk relative to others has often being used in the literature as a measure of optimism (Weinstein, 1980). For regressions, mediations and graphs we reverse scored participants’ responses such that high numbers will indicate high private optimism and low numbers low private optimism (that is a 1 became a 5, a 2 became 4 and so on).

**Public Pessimism.** Participants were asked to indicate: “Do you think COVID-19 presents a real danger to the health of the human population?” using continuous visual analogue scale from 0 (not really) to 100 (extreme danger). High numbers indicate public pessimism.

**Compliance measures.** Participants rated on a continuous visual analogue scale ranging from 0 (none at all) to 100 (a lot): a) “How much effort do you make to wash your hands regularly?”; b) “How much effort do you make to socially distance yourself from others?”; c) “How much effort do you make to avoid touching your face?”, as well on a continuous visual analogue scale ranging from 0 (zero) to 100 (many times) d) “In the past week how many times have you been to another person's house ?”; e) How many days this week have you been closer than 1 meter to another person (except those you live with)?”. Items d) and e) were reverse coded to compute a mean score of compliance.

**Sense of agency.** Participants completed a questionnaire assessing sense of agency(Lachman & Weaver, 1998).
